# Anatomy of urethro-vesical segment and vaginal microbiota: is there any connection at stress urinary incontinence?

**DOI:** 10.3389/fmed.2026.1915642

**Published:** 2026-09-29

**Authors:** Yermek M. Turgunov, Leonid V. Sevastyanov, Alyona V. Lavrinenko, Yelena V. Avilova

**Affiliations:** 1 Department of surgical diseases, Karaganda State Medical University, Karaganda, Kazakhstan; 2 Department of urology, pediatric and plastic surgery, Karaganda State Medical University, Karaganda, Kazakhstan; 3 Scientific research laboratory, Karaganda State Medical University, Karaganda, Kazakhstan; 4 LLP Alanda Clinic, Karaganda, Kazakhstan

**Keywords:** sling surgery, stress urinary incontinance, ultrasound, urethrovesical segment, vaginal microbiota

## Abstract

**Background:**

Stress urinary incontinence (SUI) is one of the most prevalent pelvic floor disorders in women. While previous studies have demonstrated associations between urinary incontinence and changes in vaginal microbial composition, the relationship between vaginal microbiota and anatomical characteristics of the lower urinary tract remains insufficiently investigated.

**Objective:**

To evaluate the association between vaginal microbiota composition and sonographic parameters of the urethrovesical segment in women with SUI before and after surgery.

**Methods:**

This study included 100 women with SUI. Ultrasound assessment included measurements of urethral length and diameter, bladder neck mobility, *α* and *β* angles, and the internal bladder angle at rest and during the Valsalva maneuver. Vaginal samples were cultured and analyzed using MALDI-TOF mass spectrometry for species identification. All patients underwent transobturator midurethral sling surgery. Follow-up examinations were performed one month postoperatively.

**Results:**

Prior to surgery, 238 microbial strains representing 49 species were isolated. Gram-positive microorganisms predominated, with Staphylococcus spp. (32.8%), Enterococcus spp. (19.8%), and Lactobacillus spp. (13.0%) being the most common taxa. Following surgery, the proportion of Lactobacillus spp. increased significantly from 13.0% to 33.5% (*p*=0.0001), while Staphylococcus spp. and Enterococcus spp. decreased significantly (*p*=0.007 and *p*=0.030, respectively). Overall bacterial load was also reduced (*p*=0.037). Significant clinical improvement was observed, reflected by marked reductions in ICIQ-SF, UDI-6, and OABSS scores. Correlation analysis demonstrated significant associations between vaginal microbiota and urethrovesical anatomy. Longer urethral length was associated with gram-positive flora and staphylococcal colonization, whereas gram-negative microorganisms, particularly Enterobacterales, were associated with shorter urethral length and smaller urethral diameter. Enterobacterial colonization was linked to a 12.1% narrower urethra (*p*=0.003). Additional associations were identified between specific bacterial groups and dynamic changes in *β*-angle and internal bladder angle measurements.

**Conclusions:**

Surgical treatment of SUI is associated with significant symptom improvement and early changes in the structure of the cultivable vaginal microbiota, including an increased representation of Lactobacillus spp. The observed associations between characteristics of the vaginal microbiota and sonographic parameters of the urethrovesical segment suggest a possible relationship between the anatomical and functional characteristics of the lower urinary tract and the local microbial environment. However, the nature of these associations and their causal direction remain undetermined. The findings should therefore be considered hypothesis-generating and require confirmation in prospective studies with a control group, longer follow-up, and the use of modern microbiome profiling methods.

## Introduction

Stress urinary incontinence (SUI) is one of the most popular pelvic floor diseases among women, accompanied with both malformation of anatomic and functional status of lower urinary tract, and composition change of urogenital microflora (1–4). In recent years it was found that peculiarities of vaginal microflora, characterized with decreased prominence of Lactobacillus spp. and increased share of opportunistic pathogens are often found among female patients with different forms of urinary incontinence (5–7). These changes are associated with symptom load and quality of patients' life (8).

Number of studies showed that development of SUI is related to the changes in anatomy of urethro-vesical segment, including the hypermobility of urinary bladder neck, abnormality of middle third of urethra support, change of angular parameters of urethro-vesical segment (9–12). However, mechanisms of correlation of structural changes in lower urinary tract with condition of urogenital microbiome remain underexplored.

The relationship between the vaginal and urinary microbiomes is of particular interest due to their anatomical proximity and the exchange of microbial communities between urogenital niches (13, 14). In both biotopes, bacteria of the genus *Lactobacillus* predominate, comprising six distinct species: *L. iners, L. crispatus, L. gasseri, L. jensenii, L. taiwanensis,* and *L. ultanensis.* Partial overlap has also been reported for several genera, including *Streptococcus, Prevotella, Ureaplasma, Shuttleworthia, Escherichia, Veillonella, Sneathia,* and *Gardnerella* (15). Moreover, some evidence suggests that the urethra may represent a distinct microbial niche (16) and thus may influence the composition of the microbiota throughout the urogenital region. A detailed comparison of urinary tract microbiota with microbiota from different sites of the female reproductive system demonstrated that the urinary tract microbiota more closely resembles the microbiota of the cervix and uterine cavity than that of the vagina in the same women (17), further highlighting the complexity of the organization, distribution, and interactions of microbial communities within the urogenital region.

In women with urinary incontinence, statistically significant associations have been identified between the composition of the urinary and vaginal microbiomes, as well as between microbiota composition and surgical treatment outcomes (18). However, data on a possible relationship between the microbiota and the anatomical and functional characteristics of the urethra, urethrovesical segment, and urinary bladder remain scarce.

The aim of this study was to identify possible associations between the qualitative composition of the ***cultivable*** vaginal microbiota and sonographic parameters of the urethrovesical segment in women with SUI, in order to gain a broader understanding of the pathogenesis of the condition and the factors influencing the outcomes of surgical treatment.

## Materials and methods

The study was performed on the basis of private medical center of Karaganda city, Kazakhstan. The work was fulfilled within the frameworks of grant by Ministry of Science and Higher Education of the Republic of Kazakhstan (URN AP23488848). The study covered 100 female patients with confirmed diagnosis of SUI. The average age amounted 51,9 ± 11,6 years old (Me 51,5; IQR: 43,0–58,0). Average body mass of patients amounted 76,4 ± 16,0 kg (Me 73,0; IQR: 68,0–82,3), BMI — 28,3 ± 4,6 kg/m^2^ (Me 27,5; IQR: 24,9–31,4), which conforms with the category of excess body weight in cohort under research. Symptoms duration varied from 1 year to 30 years 30 years and in average amounted 6,4 ± 5,5 years (Me 5,0; IQR: 3,0–10,0). The median number of pregnancies was 4 (IQR: 2.75–5), and the median number of births was 2 (IQR: 2–3).

The diagnostic algorithm included assessment of complaints and medical history, physical examination, a stress test, and completion of standardized questionnaires: the ICIQ-SF for assessment of symptom severity, the OABSS for identification of symptoms of urinary urgency, and the UDI-6 (19–21) for assessment of quality of life. Inclusion criteria were parous women aged 28–86 years with moderate to severe SUI. Exclusion criteria included abnormal urinalysis, acute inflammatory diseases of the genital tract, pelvic organ prolapse, previous surgical interventions involving the vagina, perineum, or pelvic organs, a history of malignancy, and a post-void residual urine volume >100 mL, as a potential indicator of impaired bladder emptying that could affect the functional parameters of the lower urinary tract. Patients receiving hormonal or antibacterial medications or vaginal suppositories, as well as those who had sexual intercourse within 1 month before or after surgery, were also excluded from the study.

Sonographic assessment of the anatomical structures was performed with the patient lying on the examination table and was performed by the same specialist in all cases using a Samsung Hera W10 scanner (Samsung Medison Co. Ltd., South Korea). The abdominal wall examination was performed with a 1–6 mHz convex transducer, the transvaginal examination with a 7–11 mHz cavity transducer, and the perineal examination with a 7–13 mHz linear transducer. With a bladder filling of 200–300 mL, the presence of urination urge, and a positive cough test, the following parameters were assessed: urethral length, urethral diameter, internal angle of urine bladder, *α* angle, *β* angle at rest and during the Valsalva maneuver, distance from the symphysis to the middle third of urethra and urethro-vesical segment at rest and during the Valsalva maneuver, and determination of residual urine volume before and 1 month after surgery. The distance from the pubic symphysis and urethro-vesical segment to the sling and the position of the sling relative to the length of the urethra before and 1 month after surgery were also measured.

Surgical treatment was performed using the TVT-O technique (de Leval, 2003). In all cases, an adjustable sling was implanted. Tension adjustments, if necessary, were made the day after surgery based on a comprehensive assessment of the patient's subjective sensations during urination, residual urine output, and uroflowmetry data.

To assess the ***cultivable*** vaginal microbiota, vaginal discharge samples were collected from all participants. Samples were obtained using sterile swabs with the patient in the supine position after insertion of a vaginal speculum; sampling was performed from the posterior vaginal fornix. The swab was placed in a transport tube containing Amies transport medium. All samples were delivered to the laboratory within 2 h of collection.

### Bacteriological analysis

The samples were sent to the research laboratory of the Karaganda Medical University. The culture was performed on blood agar plates supplemented with 5% sheep blood (Thermo Scientific, USA), as well as on Candida Medium (Himedia, India) and Lactobacillus MRS agar (Himedia, India). The plates were incubated for 24–48 h at 37 °C under microaerophilic conditions. Species identification of the obtained isolates was performed by matrix-associated laser desorption/ionization with transit time (MALDI-TOF MS) using the Microflex LT system and MALDI Biotyper Compass 4.1.80 software (Bruker Daltonics, Germany). The recommended score value of  ≥ 2.2 was used as a criterion for reliable species identification [36]. Additionally, a quantitative assessment of the cultivated microorganisms (CFU/mL) was carried out.

The study was approved by local ethics committee of “Karaganda Medical University” NCJSC and conducted in accordance with the World Medical Association Declaration of Helsinki, which sets forth ethical principles for medical research involving human. All participants signed written informed consent prior to inclusion into the study.

Statistical analysis was performed using IBM SPSS Statistics v.23.0 software. Quantitative data are presented as median and interquartile range; comparisons between groups were made using the Mann–Whitney test and Kruskal–Wallis test. Semiquantitative CFU values were analyzed using the Mann–Whitney U test. Pre- and postoperative changes within groups were assessed using the Wilcoxon rank test. Qualitative variables were compared using Pearson's *χ*^2^ test or Fisher's exact test (for expected frequencies less than 5). The correlation between different factors was assessed using Spearman's correlation coefficient. Statistical significance was accepted at *p* < 0.05. Correlations between the various factors were assessed using Spearman's correlation coefficient, ***with correction for multiple comparisons using the Benjamini–Hochberg false discovery rate (FDR) method.*** Statistical significance was set at *p* < 0.05.

## Results

A total of 238 cultivable isolates belonging to 49 taxonomic units were isolated from vaginal swabs collected before surgery. The mean number of microbial species per patient was 2.38 (range, 1–5). Of these isolates, 182 (76.47%) were Gram-positive bacteria, 41 (18.06%) were Gram-negative bacteria, and 7 (2.94%) were *Candida* spp. The predominant microorganisms were *Staphylococcus* spp. (78 isolates, 32.77%), *Lactobacillus* spp. (31 isolates, 13.03%), and *Enterococcaceae* (47 isolates, 19.75%). Gram-negative microorganisms of the order *Enterobacterales* accounted for 46 isolates (19.33%) and included representatives of the genera *Citrobacter, Enterobacter, Escherichia, Klebsiella, Morganella,* and *Proteus*. Among these, *Escherichia coli* was predominant, accounting for 35 strains (76.09% of all Enterobacterales isolates).

After surgery, the mean number of microbial species per patient decreased by 4.8% to 2.27 (range, 1–5). A total of 227 isolates were recovered, including 178 (78.41%) Gram-positive bacteria, 41 (18.06%) Gram-negative bacteria, and 8 (3.52%) *Candida* spp. At the same time, changes in the microbial community structure were observed. The proportion of *Lactobacillus* spp. increased significantly by 20.45% (*p* = 0.0001), whereas the proportions of *Staphylococcus* spp. and *Enterococcus* spp. decreased significantly by 11.2% and 7.42%, respectively. Microbial burden also decreased significantly: the mean decimal logarithm of the number of colony-forming units per milliliter (log10 CFU/mL) decreased from 5.3 before surgery to 4.8 after surgery (*p* = 0.037) (Table 1).

**Table 1:** Microbial profile of vaginal discharge before and after surgery.

Genus of microorganism	Before operation		After operation		*χ* ^2^	*p*
	abs	%^a^	abs	%^a^		
*Staphylococcus* spp.	78	32,77	49	21,59	7,325	**0.007**
*Streptococcus* spp.	22	9,24	20	8,81	0.027	0.871
*Enterococcus* spp.	47	19,75	28	12,33	4,72	**0.030**
*Lactobacillus* spp.	**31**	**13,03**	**76**	**33,48**	**27.44**	**0.0001**
*Enterobacteralis*	46	19,33	40	17,62	0.224	0.636
*Candida* spp.	**7**	2,94	8	3,52	0.127	0.723
Others	**7**	2,94	6	2,64	0.038	0.846
Total	**238**	**100**	**227**	**100**		

a Percentages were calculated based on the total number of cultivable isolates recovered at the respective study stage, rather than on the number of patients. A single patient could have multiple isolates representing different microorganisms. ^b^The most significant data are highlighted in bold.

In patients over 60 years old, gram-negative flora is more common than in individuals under 40 years old (68.4% vs. 13.3%; *p*=0.005), with *Enterobacterales* microorganisms being detected more frequently (63.2% and 13.3%, respectively; *p*=0.014). A weak direct correlation was found between the duration of symptoms and the presence of enterococci in the vaginal microflora before surgery. With a longer duration of urinary incontinence symptoms, enterococci were detected more frequently. The duration of symptoms in the group with detected enterococci was 15% higher (*p*=0.023).

Before adjustment for multiple comparisons, the number of pregnancies was positively correlated with the detection of Staphylococcus aureus before surgery (*ρ* = 0.259; *p* = 0.009) and Escherichia coli after surgery (*ρ* = 0.240; *p* = 0.016), as well as with the post-operative abundance of E. coli (*ρ* = 0.383; *p* = 0.040). The number of deliveries was negatively correlated with the detection of Enterococcus faecalis before surgery (*ρ* = −0.207; *p* = 0.039) and with the abundance of Lactobacillus rhamnosus after surgery (*ρ* = −0.613; *p* = 0.009). However, none of these associations remained statistically significant after false discovery rate (FDR) correction.

At study of the correlation between the qualitative and quantitative composition of the microflora subject to the severity of symptoms and quality of life before surgery using the ICIQ-SF, OABSS, and UDI-6 questionnaires no significant associations were revealed. After surgery, a weak direct correlation was found between the number of gram-positive microorganism associations in a smear and the UDI-6 value (Table 2), at that, higher number of gram-positive bacterial associations in a smear leads to higher values in UDI-6 scale (*p* < 0.05).

**Table 2:** Correlation of microflora values and questionnaires after surgery.

Parameters of microflora		OABSS	ICIQ-SF	UDI-6
Total types	r	0,102	0,019	0,115
	p	0,313	0,853	0,255
Gram-positive bacteria	r	0,186	0,102	**0,280**
	p	0,064	0,314	**0,005**
Gram-negative bacteria	r	−0,124	−0,077	−0,181
	p	0,217	0,446	0,071
*Staphylococcus*	r	0,021	0,092	0,188
	p	0,838	0,363	0,061
*Streptococcus*	r	0,025	−0,044	−0,08
	p	0,804	0,664	0,427
*Enterococcus*	r	0,18	0,141	0,146
	p	0,073	0,161	0,146
*Lactobacteria*	r	0,087	−0,025	0,031
	p	0,392	0,809	0,763
*Enterobacteria*	r	−0,134	−0,089	−0,184
	p	0,183	0,377	0,067
*Candida*	r	−0,064	−0,017	−0,103
	p	0,527	0,867	0,31
Average CFU/mL	r	0,081	0,022	0,155
	p	0,424	0,824	0,124

r-Spearman's rank correlation, *p*- significant level. The most significant data are highlighted in bold.

At assessment of the dynamics of questionnaire scores after surgical treatment, a statistically and clinically significant decrease in symptoms according to the OABSS scale was observed after 1 month: the average score decreased from 5.1 ± 4.6 (Me 4.5; IQR: 0–9.0) to 0.8 ± 2.3 and (Me 0; IQR: 0.0–0.0). According to UDI-6 data, a reliable decrease in scores was noted from 10.1 ± 3.3 (Me 10.0; IQR: 8–12.0) before surgery, up to 1.0 ± 2.2 (Me 0.0; IQR: 0.0–0.0) after surgery. According to the ICIQ-SF questionnaire, a significant decrease in the symptom load was recorded: before surgery, the average score was 14.9 ± 3.7 (Me 16.0; IQR: 12.8–18.0), after surgery, a decrease in the indicator to 1.01 ± 2.96 (Me 0; (IQR: 0.0–18.0) was noted, which corresponds to the transition of most patients from the category of moderate and severe UI symptoms to minimal or subclinical manifestations.

At study of the correlation between vaginal microflora and sonographic parameters of the urethro-vesical segment before surgery, a weak direct correlation between urethral length at rest and during the Valsalva maneuver with the presence of gram-positive flora, particularly staphylococci, was disclosed. However, there was a weak inverse correlation between urethral length and diameter and gram-negative flora, particularly Enterobacteria. There was also a weak direct correlation between the posterior *β* angle at rest and the presence of staphylococci (Table 3).

**Table 3:** Correlation between sonographic readings of urethro-vesical segment and vaginal microflora before the surgery.

Sonographic readings of urethro-vesical segment	Vaginal microflora readings before the surgery							
	Spearman's correlation	Gr+ flora	Gr- flora	Staphylococci	Streptococci	Lactobacteria	Enterobacteria	Average Lg(CFU)
Urethral length at rest	r	**0,21**	**−0,19**	**0,28**	−0,09	−0,01	**−0,21**	**−0,23**
	p	**0,04**	**0,05**	**0,01**	0,38	0,91	**0,03**	**0,02**
Urethral length at Valsalva maneuver	r	**0,20**	**−0,22**	**0,21**	−0,17	0,04	**−0,22**	**−0,22**
	p	**0,05**	**0,03**	**0,04**	0,09	0,72	**0,03**	**0,03**
Urethral diameter at rest	r	0,18	**−0,21**	0,19	0,03	0,12	**−0,30**	−0,09
	p	0,07	**0,04**	0,06	0,78	0,22	**0,00**	0,35
Urethral diameter at Valsalva maneuver	r	0,13	−0,15	0,05	−0,04	**0,20**	**−0,20**	0,06
	p	0,18	0,15	0,65	0,71	**0,05**	**0,04**	0,55
Posterior *β* angle at rest	r	0,13	−0,02	**0,21**	0,01	−0,05	−0,07	−0,07
	p	0,20	0,87	**0,04**	0,95	0,61	0,49	0,46
Deviations of β angle	r	−0,14	−0,01	**−0,26**	0,04	0,09	0,03	0,03
	p	0,17	0,95	**0,01**	0,68	0,37	0,73	0,79
Deviation of internal angle of UB	r	−0,05	−0,01	0,09	**−0,23**	−0,02	−0,08	0,04
	p	0,70	0,91	0,46	**0,04**	0,88	0,47	0,75

r-Spearman's rank correlation, *p*- significant level. The most significant data are highlighted in bold.

With urethral length increases, the average Lg(CFU) level decreases, the trend line is described by the equation y=6.1-0.03x; where y is the average Lg(CFU) value of the detected vaginal microflora, and x is the urethral length according to sonography data (Figure 1). Thus, a weak negative correlation was found between urethral length at rest before surgery and the level of vaginal bacterial contamination (R^2^=0.059); a similar correlation also persists with the Valsalva maneuver.

**Figure 1:**
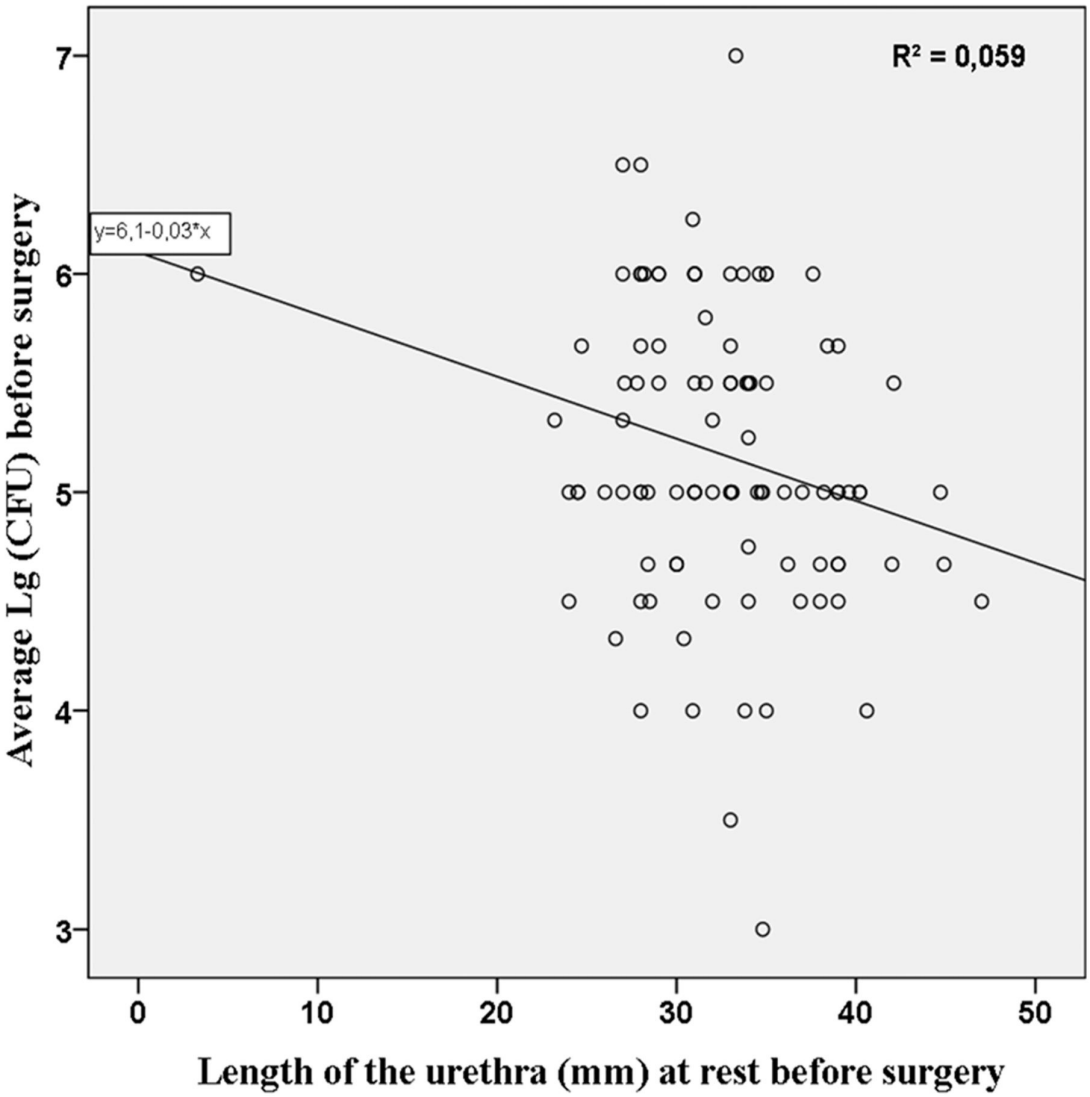
Correlation between urethral length at rest before surgery and average quantity of CFU.

In the group with staphylococci detected in vaginal secretion, the urethra was on average 3.25 mm (10.7%) longer (*p*=0.008), that is, the appearance of staphylococci in the microflora is associated with the length of the urethra.

In the group of patients with detected enterobacteria in the vagina before surgery, the urethral diameter was on average 1.1 mm (12.1% smaller, *p*=0.003), and the same trend was observed with the Valsalva maneuver (by 10%, *p*=0.05). This means that the presence of enterobacteria in the vaginal microflora is associated with a narrower urethra. Whilst wider urethral diameter is associated with the presence of lactobacteria in the microflora (*p*=0.055).

The presence of staphylococci in the vaginal microflora is associated with a decrease in the angle during the Valsalva maneuver (*p*=0.027) (Figure 2). Negative values indicate a decrease in the angle during the maneuver, while positive values indicate an increase. The majority of negative values is represented by patients with positive staphylococcal contamination.

**Figure 2:**
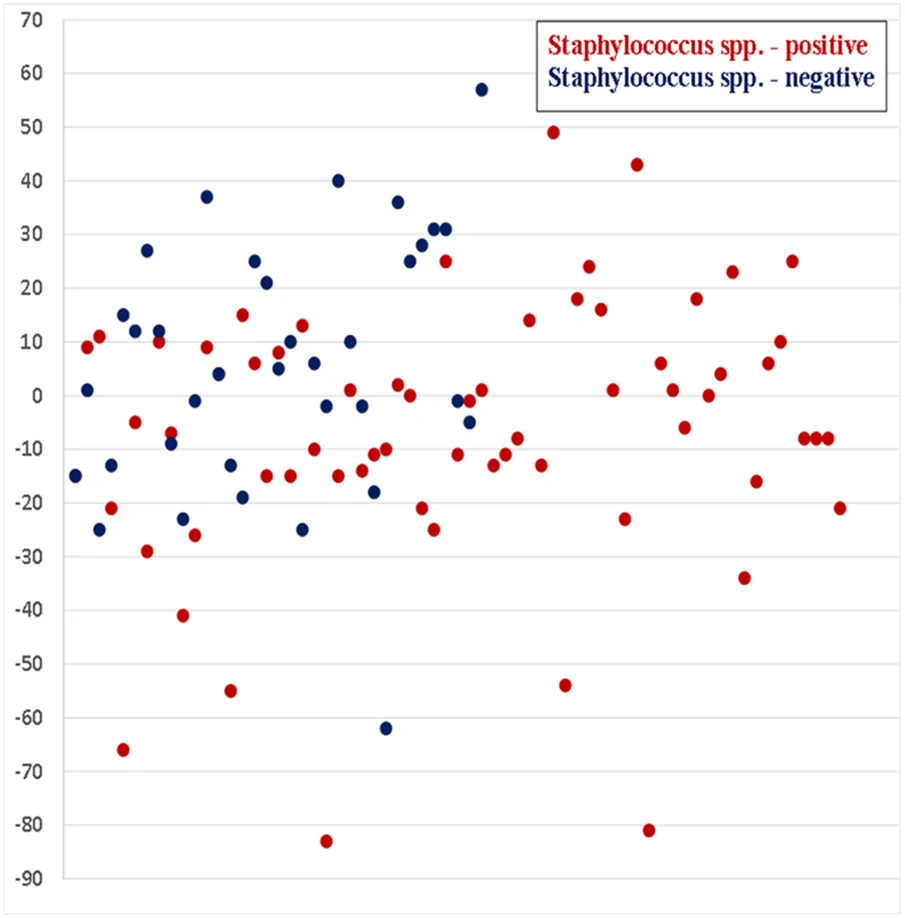
Distribution of angle *β* deviations (degrees).

A decrease in the internal angle of urinal bladder during the Valsalva maneuver is associated with the presence of streptococci in the vaginal microflora (*p*=0.044) (Figure 3).

**Figure 3:**
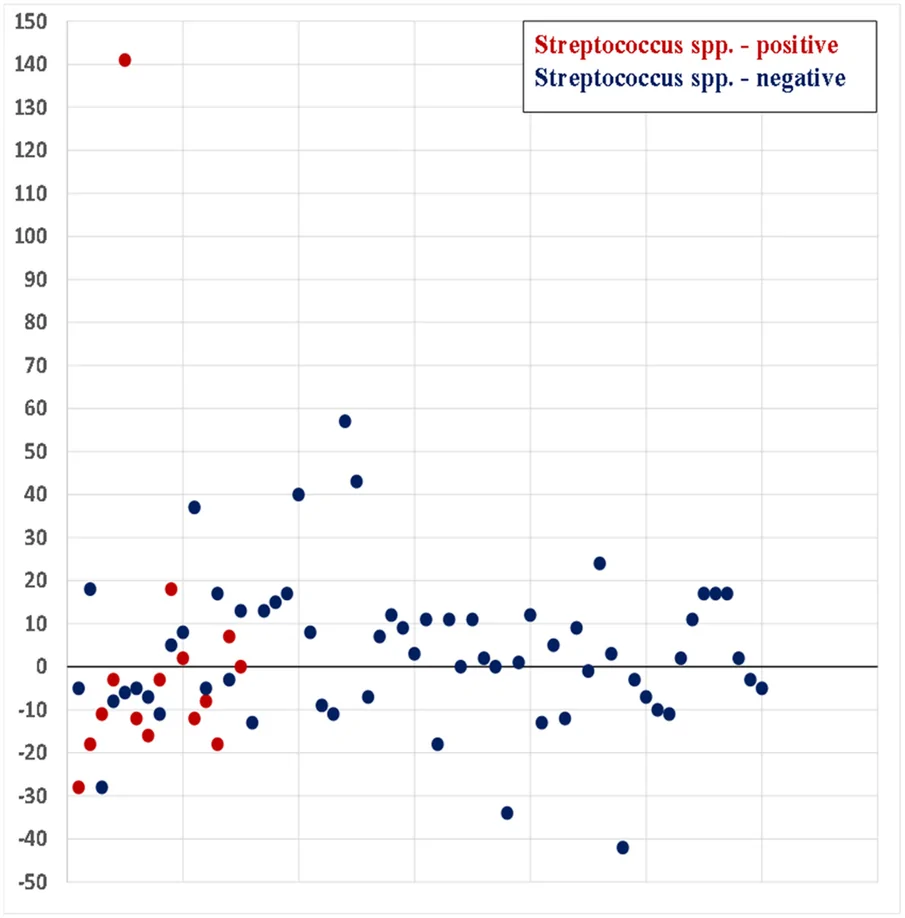
Diagram of UB internal angle deviations distribution (degrees).

Negative values indicate a decrease in the angle during the maneuver, while positive values indicate an increase. The majority of positive streptococcal cultures are found in the negative range.

The study of the relationship between ultrasound parameters and vaginal microflora after surgery revealed a direct correlation between the length and diameter of the urethra and the vaginal microflora colonization index (Figures 4, 5) and an inverse correlation with the presence of streptococci. Direct relationship: between the internal angle of the bladder and the presence of gram-negative flora, in particular enterobacteria. Inverse relationship: between the deviation of the *α* angle and the presence of streptococci in a vaginal smear—the deviation of the alpha angle during the Valsalva maneuver after surgery was, on average, 2.6 times smaller (*p*=0.017) same between the deviation of the *β* angle and the presence of staphylococci in the vaginal microflora after surgery (Table 4).

**Figure 4:**
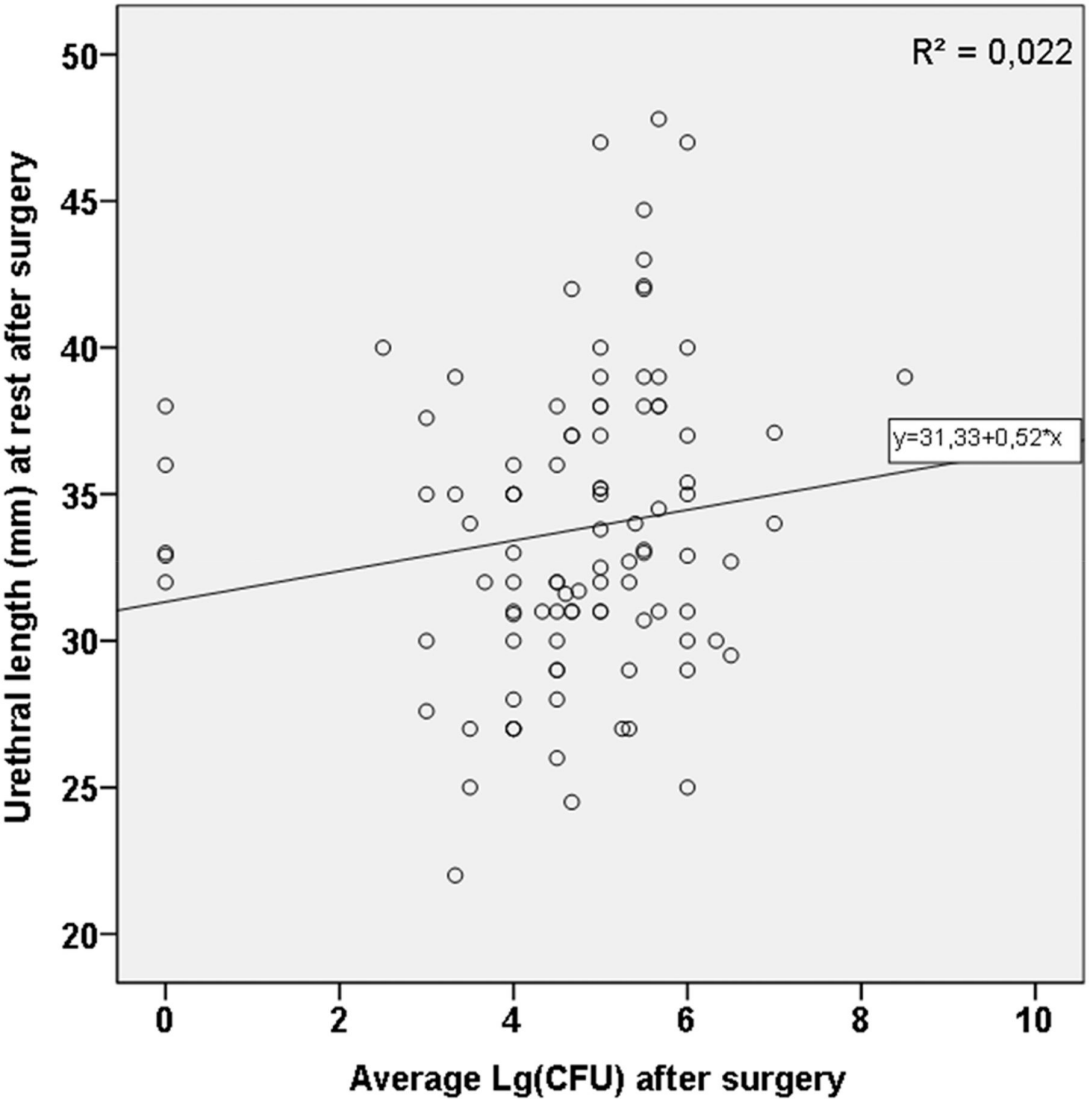
Relationship between ultrasound parameters of length of urethra with contamination level of vaginal microflora after surgery.

**Figure 5:**
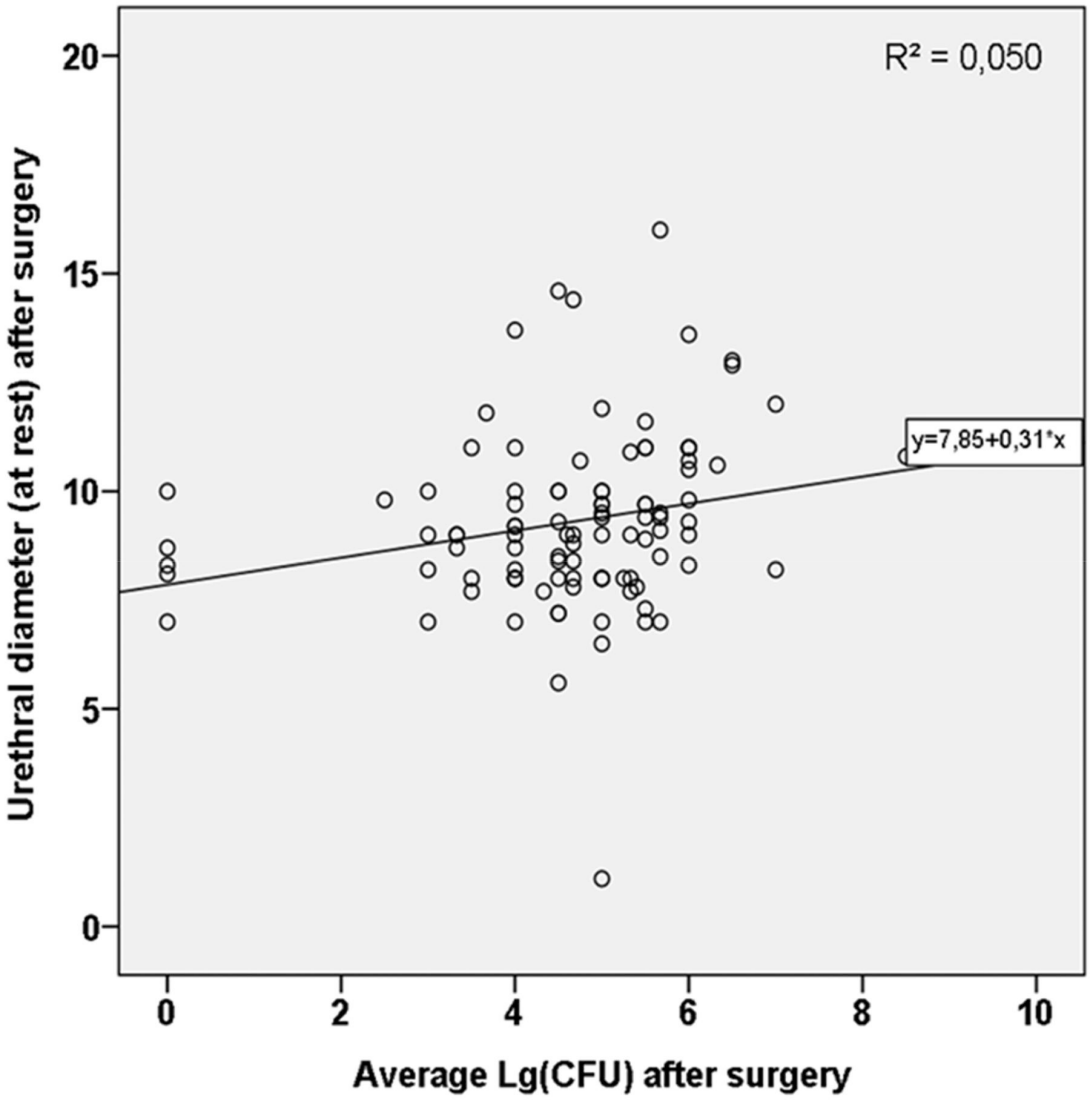
Relationship between ultrasound parameters of diameter of urethra with contamination level of vaginal microflora after surgery.

**Table 4:** Correlation of ultrasound parameters with vaginal microflora after surgery.

Sonographic readings of urethro-vesical segment after surgery		Vaginal microflora readings after the surgery				
		Average Lg(CFU)	Gr- flora	Staphylococci	Streptococci	Enterobacteria
Urethral length at rest	r	**0,221**	0,036	0,090	**−0,197**	0,050
	p	**0,027**	0,725	0,371	**0,049**	0,624
Urethral length at Valsalva maneuver	r	**0,206**	0,047	0,019	**−0,210**	0,035
	p	**0,039**	0,643	0,850	**0,036**	0,732
Urethral diameter at rest	r	**0,283**	0,129	0,114	**−0,208**	0,104
	p	**0,004**	0,202	0,259	**0,038**	0,303
Urethral diameter at Valsalva maneuver	r	**0,210**	0,059	0,122	**−0,251**	0,070
	p	0,036	0,557	0,228	0,012	0,486
Internal angle of urine bladder	r	0,007	**0,227**	0,071	0,155	**0,227**
	p	0,952	**0,034**	0,514	0,152	**0,034**
Internal angle of urine bladder at Valsalva maneuver	r	0,015	**0,214**	0,004	0,141	0,178
	p	0,893	**0,047**	0,973	0,192	0,099
Deviations of *α* angle (degrees)	r	0,15	0,11	0,05	**−0,240**	0,09
	p	0,13	0,29	0,62	**0,02**	0,37
Deviations of β angle (degrees)	r	0,02	0,07	**−0,215**	0,05	0,04
	p	0,82	0,51	**0,03**	0,61	0,70

r- Spearman's rank correlation, *p*- significant level. The most significant data are highlighted in bold.

## Discussion

The findings obtained indicate that surgical correction of urinary incontinence is accompanied not only by marked clinical improvement in the condition of the patients, but also by changes in the *culturable* vaginal microbiota. Before surgery, the vaginal microbiota was characterized by considerable variability in species composition, with a predominance of Gram-positive microorganisms, primarily staphylococci and enterococci, as well as a substantial representation of Enterobacterales. Following surgical treatment, a moderate decrease in the overall number of recovered microorganisms and in the level of bacterial burden was observed; however, the most important finding was a change in the structure of the ***cultivable*** vaginal microbiota. This was manifested by a significant increase in the proportion of *Lactobacillus spp.* accompanied by a simultaneous decrease in the frequency of detection of staphylococci and enterococci. However, this shift in the composition of the cultivable microbiota cannot be regarded as evidence of sustained normalization of the microbial community, given the short interval before postoperative reassessment.

It is known that lactobacilli are the key component of the normal vaginal microbiota and provide colonization resistance through the production of lactic acid, hydrogen peroxide, and bacteriocins. Therefore, an increase in their proportion after surgery can be considered as indirect sign of restoration of local microbial homeostasis. Given the lack of specific antibacterial correction of the microflora, the observed changes are likely related to the elimination of functional disorders of the lower urinary tract, a reduction in episodes of urinary leakage, and a reduction in chronic irritation of mucosa in the urogenital area.

Рarticular interest is attracted by the age-related dependence of the microflora composition. Gram-negative microorganisms and members of the *Enterobacterales* family were detected significantly more frequently in patients over 60 years old than in younger women. The findings are consistent with modern understanding of age-related changes in the urogenital microbiome caused by estrogen deficiency, decreased lactobacilli, and increased susceptibility to colonization by intestinal flora. This suggests that older age may be a contributing factor to the development of an unfavorable microbial profile in patients with urinary incontinence.

An interesting finding was the disclosed connection between the duration of urinary incontinence symptoms and the presence of enterococci in the vaginal microflora. Longer symptom duration was associated with a higher frequency of detection of *Enterococcus spp.* This finding may reflect the long-term chronic changes in the urogenital environment in patients with urinary incontinence and supports the hypothesis that disease duration can influence the formation of stable microbial communities.

In spite of manifested differences in the microflora composition, no significant associations were found between the microbial landscape and symptom severity according to the ICIQ-SF, OABSS, and UDI-6 scales before surgery. Probably, the severity of clinical manifestations of urinary incontinence is determined more by anatomical and functional abnormalities of the urethro-vesical segment than by characteristics of the vaginal microflora.

An important finding of the study was the identification of associations between characteristics of the ***cultivable*** vaginal microbiota and ultrasound parameters of the urethrovesical segment. Before surgery, a longer urethra was associated with a predominance of Gram-positive flora and staphylococci, whereas the presence of Gram-negative microorganisms and Enterobacterales correlated with a shorter urethral length and smaller urethral diameter. In addition, a weak inverse association was observed between urethral length and the level of vaginal bacterial burden. Although the strength of the observed correlations was low, these findings suggest a potential relationship between the anatomical characteristics of the lower urinary tract and the local microbial environment.

Of particular interest was the association between Enterobacterales and a smaller urethral diameter. Theoretically, this anatomical feature may contribute to alterations in urodynamics, impaired natural drainage of the periurethral region, and ***may be statistically associated with specific patterns of microbial colonization of the periurethral and vaginal regions.*** Conversely, the tendency toward a larger urethral diameter among patients with a predominance of lactobacilli may indirectly reflect a more favorable functional state of the urogenital tract. However, the directionality of the presumed associations remains unknown. Anatomical characteristics of the urethra may create conditions that influence microbial colonization; however, the reverse sequence cannot be excluded, whereby features of the microbial environment may be associated with changes in the local tissue state. A single baseline assessment does not allow the temporal sequence of these phenomena to be established and, consequently, does not permit conclusions regarding a causal relationship between urethral anatomical parameters and characteristics of the vaginal microbiota.

After surgery, the pattern of these associations changed. Whereas greater urethral length was associated with a lower bacterial burden before the intervention, an inverse relationship between urethral length and diameter and the level of CFU was observed postoperatively. This change in the direction of the associations is of particular interest because it occurred following a surgical intervention that modifies the anatomical and functional characteristics of the mid-urethra.

One possible explanation is that suburethral sling placement may alter the local urogenital milieu. Changes in the position and support of the mid-urethra s

## Conclusion

Overall, the findings of the present study indicate that one month after surgical treatment of stress urinary incontinence (SUI), changes in the composition of the culturable vaginal microbiota were observed, characterized by an increased proportion of Lactobacillus spp. and a decreased representation of several other culturable microorganisms. However, the short follow-up period does not allow the persistence of these changes to be determined or establish whether they reflect long-term remodeling of the vaginal microbial community. In addition, this study is the first to demonstrate statistically significant associations between the composition of the culturable vaginal microbiota and ultrasound characteristics of the urethrovesical segment. The biological direction and causal nature of these associations remain unclear and warrant further investigation. Further studies incorporating a control group and a longer follow-up period of 6–12 months are needed to confirm these findings, determine whether the observed changes persist over time, and assess whether they are associated with surgical outcomes.

## Data Availability

The raw data supporting the conclusions of this article will be made available by the authors, without undue reservation.
